# Retrospective observational cohort study of laparoscopic surgical strategies for gastrointestinal stromal tumors

**DOI:** 10.1007/s13304-024-01816-4

**Published:** 2024-04-05

**Authors:** Takeharu Enomoto, Shinya Mikami, Takehito Otsubo, Masaki Hiwatari, Yoshitsugu Tsukamoto, Yasuhito Hisatsune, Jin Shimada, Tsunehisa Matsushita

**Affiliations:** Department of Gastroenterological and General Surgery, St. Marianna University School of Medicine, 2-16-1 Sugao, Miyamae-Ku, Kawasaki, Kanagawa 216-8511 Japan

**Keywords:** Submucosal tumor, Gastrointestinal stromal tumor, Laparoscopic and endoscopic cooperative surgery, Tumor location

## Abstract

Laparoscopic surgery has been used to treat gastric submucosal tumors (SMTs). Laparoscopic and endoscopic cooperative surgery (LECS) has been used when subtotal resection has been difficult, which enabled resection of these tumors. In this study, we reviewed the medical records of patients with gastric SMTs who underwent laparoscopic surgery in our hospital with the aim of reporting the surgical indications, procedures (especially for LECS), and outcomes of surgery. This study involved 55 patients who underwent laparoscopic surgery between April 2014 and March 2021. We classified the patients into two groups: laparoscopy-assisted surgery group (non-LECS group, *n* = 30) and LECS group (*n* = 25). LECS was performed in the upper stomach, in the greater curvature of the lower stomach, and in both intraluminal and intramural locations in the middle stomach. Non-LECS was selected for extraluminal and intramural tumors in the greater curvature of the upper stomach. There were no severe complications associated with the operation. There was one postoperative complication in the LECS group. The length of postoperative hospital stay did not significantly differ between the LECS and non-LECS groups. We reported the surgical procedures for gastric SMTs in our hospital. It is essential to make full use of the multiple techniques reported in this article and examine the location of the tumor to avoid excess or insufficient resection. Our review of the present case series allowed us to select the appropriate surgical approach for gastric SMTs based on the lesion location and type of development.

## Introduction

Recently, laparoscopic surgery for gastric cancer has progressed as a specialized surgical technique owing to the standardization of the methodology and evaluation of the outcomes. Laparoscopic surgery has been used to treat gastric submucosal tumors (SMTs), especially gastrointestinal stromal tumors (GISTs). In cases where subtotal resection is challenging, laparoscopic and endoscopic cooperative surgery (LECS) has been performed to enable the resection of these tumors [1]. Several LECS techniques have been introduced, but no report has acknowledged which method should be chosen for which lesion.

The aim of this study was to report the surgical indications, procedures (especially for LECS), and outcomes of the resection of gastric SMTs. Based on a review of 55 cases, we recommended the appropriate technique based on the tumor location.

## Methods

### Reporting guidelines

This retrospective observational cohort study was performed in accordance with the STROBE reporting guidelines.

### Patients

**Fig. 1:**
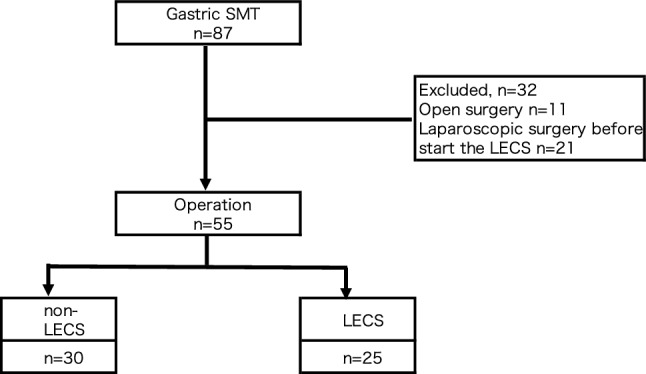
Flowchart of patient inclusion and exclusion

Eighty-seven patients underwent surgery for gastric SMTs at St. Marianna University School of Medicine Hospital between January 2010 and March 2021. We started performing laparoscopic surgery (non-LECS), as well as open surgery, in 2010. Subsequently, LECS has been performed since 2014. Fifty-five patients underwent laparoscopic surgery between April 2014 and March 2021 after LECS was introduced and were included in this study. We classified the patients into two groups: the laparoscopy-assisted surgery group (non-LECS group, *n* = 30) and the LECS group (*n* = 25) (Fig. 1).

### Indications

Preoperative upper gastrointestinal endoscopy and abdominal enhanced computed tomography with a foaming agent were performed to localize the tumor in the stomach and determine the extent of tumor development. In both groups, the tumor was located in the stomach, and tumors were categorized as intraluminal, extraluminal, or intramural. The surgical method was selected by considering the tumor size and location. Since April 2014, LECS has become a standard procedure in our hospital. For the extraluminal tumor type, we perform laparoscopic surgery. For intraluminal and intramural tumors measuring ≤ 30 mm in size, non-exposed endoscopic wall-inversion surgery or non-LECS surgery is the established procedure. For tumors larger than 30 mm in diameter, we perform LECS.

### Procedures

**Fig. 2:**
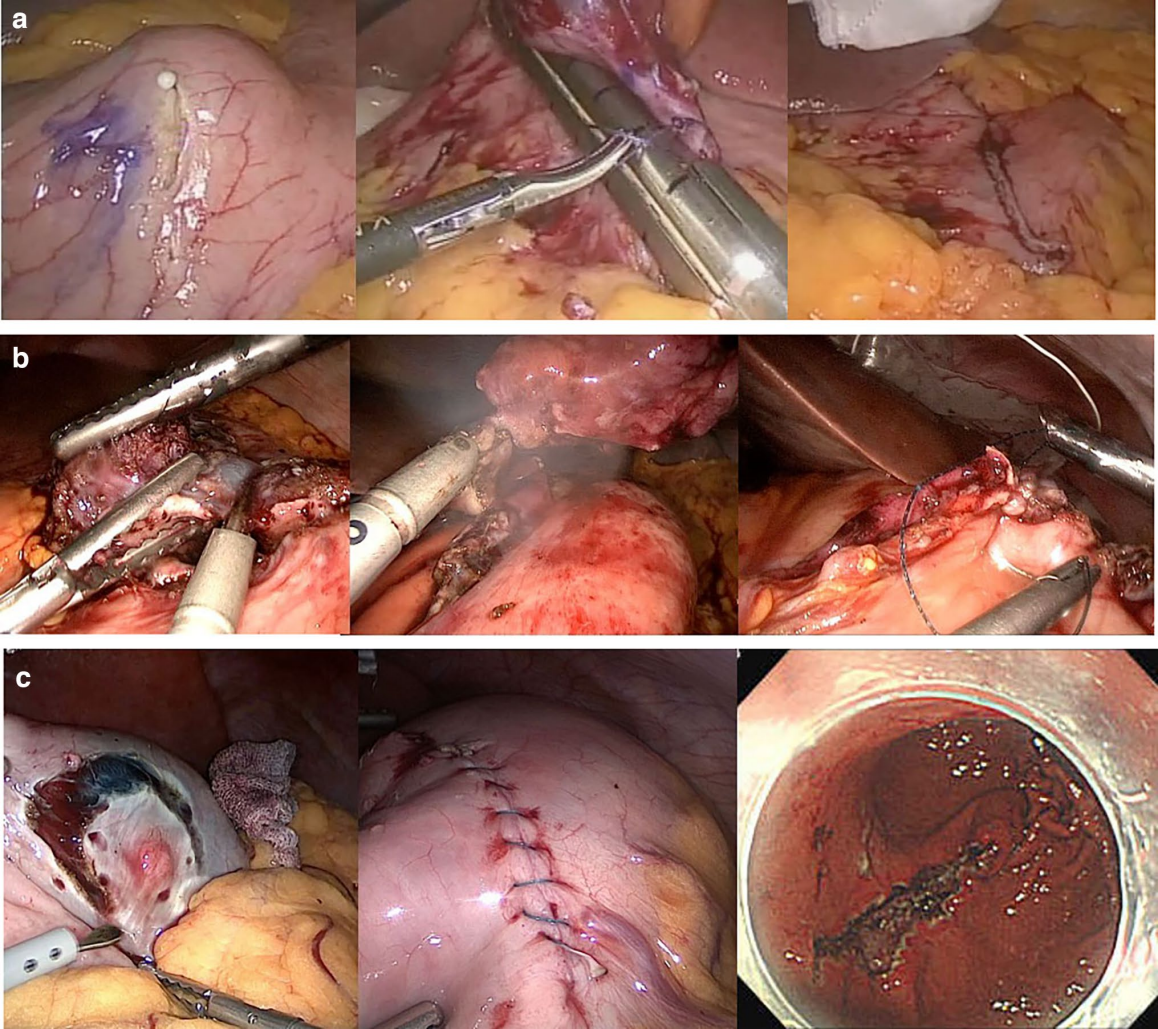
Procedures performed in the LECS group. **a** Semi-circumferential incision + automatic suturing using a stapler. **b** Circumferential resection + suture closure. **c** Non-exposed endoscopic wall-inversion surgery (NEWS). *LECS* laparoscopic and endoscopic cooperative surgery

We performed laparoscopic partial resection using a stapler, laparoscopic-assisted partial resection (open surgery-assisted resection and reconstruction), and laparoscopic-assisted proximal gastrectomy in the non-LECS group. In the LECS group, four procedures were performed:

1. Semi-circumferential incision + automatic suturing using a stapler (Fig. 2a)

After rotating the lesion toward the abdominal cavity, we inserted three needles with 3–0 absorbable suture attached to support the tumor. We then resected the lesion using an automatic suture device (stapler) and placed the excised tissue in a specimen bag to remove the lesion from the abdominal cavity. We sutured the seromuscular layer using the same 3–0 absorbable sutures. We use this method for intraluminal and intramural tumors measuring > 30 mm in diameter. This method was introduced early in the study period.

2. Circumferential resection + suture closing: simple closure (Fig. 2b)

We resected the lesion using LECS and placed the excised tissues in a specimen bag to remove the lesion from the abdominal cavity. We then performed full-thickness and seromuscular suturing with 3–0 absorbable sutures. We perform this method for intraluminal and intramural tumors measuring >30 mm in diameter. 

These two methods comprised LECS in the broadest sense.

3. Non-exposed endoscopic wall-inversion surgery [2] (Fig. 2c)

We first used an endoscope to mark the mucosal surface around the lesion. Then, under endoscopic navigation, we incised the serosal surface circumferentially around the lesion. The seromuscular layer was then sutured in a straight line by hand, and laparoscopically, the lesion was turned toward the lumen using a surgical sponge. Finally, an endoscope was used to make a circumferential incision in the mucosa around the lesion, and the submucosal layer was incised to remove the implanted sponge and the lesion. We use this method for intraluminal and intramural tumors measuring ≤ 30 mm in diameter.

4. Combined laparoscopic and endoscopic approaches to neoplasia with a non-exposure technique: CLEAN-NET [3]

The serosa was marked circumferentially around the lesion using a laparoscope. An endoscope was then used to retract the mucosa around the lesion. After confirming that the distance between the lesion and the stomach wall was sufficiently secured by stretching the mucosa, a linear stapler was used to close the mucosal base, the incised seromuscular layer was sutured in a straight line, and the lesion was removed through the abdomen using a specimen bag. We use this method for intraluminal and intramural tumors measuring > 30 mm in diameter.

### Measurement parameters

We recorded the values for the following parameters: patient age, patient sex, tumor size, preoperative diagnosis of GIST, final pathological diagnosis, tumor location [4], type of tumor growth, operative procedure, operative time, blood loss volume, complications, and postoperative length of stay.

### Statistical analysis

All statistical analyses were performed using JMP Pro 16.2.0 (SAS Institute Inc., Cary, NC, USA). All tests were two-sided, with a significance level of *p* = 0.05. The demographic characteristics of the two groups were compared using a two-sample independent t test and Fisher’s exact test. Data are presented as mean ± standard deviation.

## Results

The patients’ characteristics are shown in Table 1. The mean age of the patients in the non-LECS group was 69 (47–84) years, and the male/female ratio was 13/17. The mean tumor size was 36 (20–70) mm. The mean age of the patients in the LECS group was 64 (36–79) years, and the male/female ratio was 10/15. The mean tumor size was 24 (10–40) mm.

**Table 1:** Characteristics of 55 patients who underwent surgery for gastric submucosal tumors

		Non-LECS group (*n* = 30)	LECS group (*n* = 25)
Age	Mean ± SD (range), years	69 ± 11 (47–84)	64 ± 14 (36–79)
Sex	Male/female	13/17	10/15
Tumor size	Mean ± SD (range), mm	36 ± 16 (20–70)	24 ± 7 (10–40)
Preoperative diagnosis of GIST		5 (16.7%)	12 (48%)
Final diagnosis	GIST (low)	23	20
	GIST (intermediate)	5	0
	GIST (high)	2	0
	Leiomyoma	0	4
	Inflammatory polyp	0	1
Tumor location	E	0	2
	U	26	15
	M	4	7
	L	0	1
Type of tumor growth	Intraluminal (a)	7	17
	Extraluminal (b)	14	2
	Intramural (c)	9	6
Type of tumor growth and tumor location	E-intraluminal	0	1
	E-intramural	0	1
	U-intraluminal	7	12
	U-extraluminal	10	2
	U-intramural	9	1
	M-intraluminal	0	3
	M-extraluminal	4	0
	M-intramural	0	4
	L-intraluminal	0	0
	L-extraluminal	0	0
	L-intramural	0	1

	U-intraluminal	(*n* = 7)	(*n* = 12)
	Ant	0	0
	Post	1	1
	Les	1	6
	Gre	5	5
Tumor location and type of tumor growth	E, Ant	0	1 (a0b0c1)
	E, Post	0	1 (a1b0c0)
	U, Ant	5 (a0b2c3)	0
	U, Post	1 (a1b0c0)	1 (a1b0c0)
	U, Les	6 (a1b4c1)	9 (a6b2c1)
	U, Gre	14 (a5b4c5)	5 (a5b0c0)
	M, Ant	0	2 (a0b0c2)
	M, Les	3 (a0b3c0)	3 (a1b0c2)
	M, Gre	1 (a0b1c0)	2 (a2b0c0)
	L, Post	0	1 (a1b0c0)

Tumor designation: *E* esophagogastric junction, *U* upper third of the stomach, *M* middle third of the stomach, *L* lower third of the stomach. *Ant* anterior, *GIST* gastrointestinal stromal tumor, *Gre* greater curvature, *high* high risk, *LECS* laparoscopic and endoscopic cooperative surgery, *Les* lesser curvature, *low* low risk, *intermediate* intermediate risk, *Post* posterior, *SD* standard deviation

A preoperative diagnosis of GIST was made in 5/30 (16.7%) of the patients in the non-LECS group and 12/25 (48%) in the LECS group. The final diagnosis was low-risk GIST in 23 patients, intermediate-risk GIST in 5 patients, and high-risk GIST in 2 patients in the non-LECS group, and low-risk GIST in 20 patients, leiomyoma in 4 patients, and inflammatory polyp in 1 patient in the LECS group.

SMTs were most commonly found in the upper third (22) and middle third (27) of the stomach. There were 15 lesser curvature tumors and 5 anterior wall tumors found in the upper third of the stomach, and 19 greater curvature tumors and 6 lesser curvature tumors found in the middle third of the stomach, with the lesser and greater curvatures being the most common locations.

Intraluminal and intramural lesions were common in the upper third and middle third of the stomach. In the esophagogastric junction and lower third of the stomach, tumor growth was predominantly intraluminal and intramural. However, most tumors in the upper third of the stomach were extraluminal lesions, and extraluminal lesions were also observed in the middle third of the stomach.

Table 2 shows the clinical outcomes after surgery in each group. Operative procedures in the non-LECS group comprised laparoscopic partial resection in 26 patients, laparoscopic-assisted partial resection in 3 patients, and laparoscopic-assisted proximal gastrectomy in 1 patient. The mean operative time was 150 ± 82 min, and the mean blood loss volume was 56 ± 136 ml. Two postoperative complications occurred: postoperative pancreatic fistula in one patient and urinary tract infection in another. The mean postoperative length of stay was 9 ± 9 days.

**Table 2:** Outcomes of laparoscopic surgery in the non-LECS and LECS groups

		Non-LECS group (*n* = 30)	LECS group (*n* = 25)	*p* values
Operative procedure		LPR/ LAPR//LAPG 26/3/1	Stapler/Simple closure/ NEWS/CLEAN-NET 3/13/3/1	–
Operative time	Mean ± SD (range), min	150 ± 82 (53–386)	188 ± 63 (99–285)	< 0.01
Blood loss volume	Mean ± SD (range), ml	56 ± 136 (5–634)	47 ± 85 (5–301)	0.97
Complications		Postoperative pancreatic fistula: *n* = 1 Urinary tract infection: *n* = 1	Abdominal abscess: *n* = 1	1.0
Postoperative length of stay	Mean ± SD (range), days	9 ± 9 (4–15)	11 ± 12 (5–57)	0.11

*CLEAN-NET* combination of laparoscopic and endoscopic approaches to neoplasia with non-exposure technique, *LAPG* laparoscopic-assisted proximal gastrectomy, *LAPR* laparoscopic-assisted partial resection, *LECS* laparoscopic and endoscopic cooperative surgery, *LPR* laparoscopic partial resection, *NEWS* non-exposed endoscopic wall-inversion surgery, *SD* standard deviation

Operative procedures in the LECS group comprised stapling in 3 cases, simple closure in 13 cases, non-exposed endoscopic wall-inversion surgery in 3 cases, and CLEAN-NET in 1 case. The mean operative time was 188 ± 63 min, and the mean blood loss volume was 47 ± 85 ml. One complication was encountered: an abdominal abscess developed in one patient. The mean postoperative length of stay was 11 ± 12 days.

**Fig. 3:**
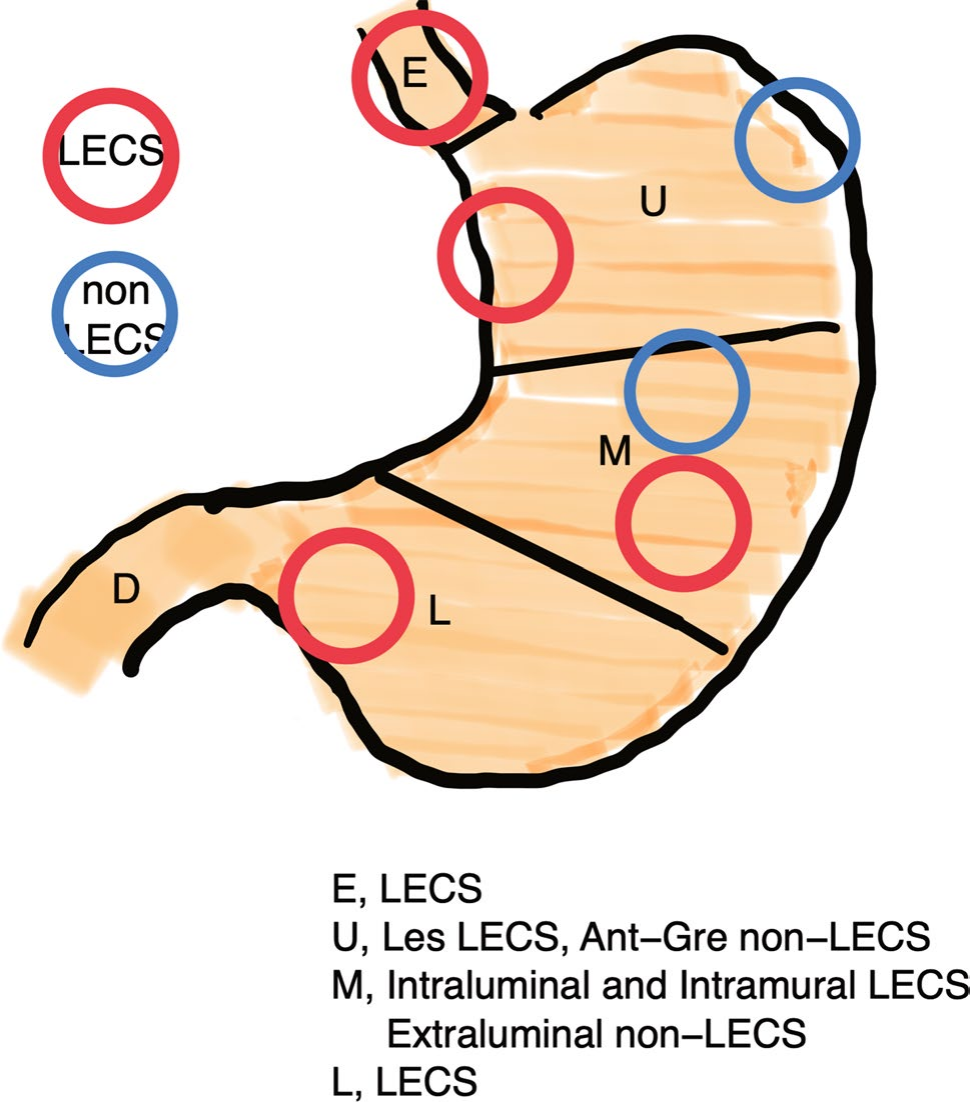
Strategy for choosing the type of surgery for laparoscopic resection of gastric submucosal tumors. *D*, duodenum, *E* esophagus, *L* lower third of the stomach, *LECS* laparoscopic and endoscopic cooperative surgery;* M* middle third of the stomach,* U* upper third of the stomach

Figure 3 shows the strategy for selecting the type of surgery, in accordance with our data. Gastric SMTs in the esophagogastric junction are treated with LECS; tumors in the lesser curvature in the upper third of the stomach are treated with LECS; tumors in the anterior wall and greater curvature are treated with non-LECS; extraluminal lesions in the middle third of the stomach are treated with non-LECS; intraluminal and intramural lesions in the middle third of the stomach must be treated with LECS; tumors in the lower third of the stomach are treated with LECS.

## Discussion

In this study, we divided the patients into two groups, the non-LECS (laparoscopic surgery) group and the LECS group, to determine the clinical characteristics of surgery for gastric SMTs. We examined the types of lesions within the LECS group. We found a smaller tumor size in the LECS group compared with the non-LECS group. This may be because, recently, needle biopsies have been performed more aggressively for small gastric SMTs, and patients diagnosed with GISTs using this method were included in the non-LECS group. We also examined the relationship between the location of the lesion in the stomach and the tumor type. As shown in Table 1, LECS was performed for lesions of the esophagogastric junction and the lower stomach. This was owing to the narrow lumen of the stomach in these locations. For the middle stomach, non-LECS was selected in all cases for extraluminal lesions, while LECS was selected for intraluminal and intramural lesions.

Current guidelines recommend surgery for resectable gastrointestinal SMTs and local resection of the gastrointestinal tract with negative margins, with confirmation that no injury to the tumor has occurred. Systematic lymph node dissection is not recommended during surgery because of the uncertain efficacy [5]. The usefulness of a simultaneous laparoscopic and endoscopic technique for resection of gastrointestinal SMTs was first reported in 2008 by Hiki et al. [1], and since then, other methods have been reported [2, 3]. We introduced laparoscopic-assisted distal gastrectomy and total gastrectomy for gastric cancer in our hospital in the same year [6]. We also began treating gastric SMTs using laparoscopic surgical techniques developed for gastric cancer.

LECS was used for the first time in our hospital in 2014 for early-stage esophagogastric junction cancer in an older, high-risk patient. After a presentation at a multidisciplinary cancer board meeting, we obtained approval from the St. Marianna University School of Medicine Ethics Committee (approval number 2609) to perform LECS. In Japan, LECS has been covered by national medical insurance since April 2014, and we have been actively performing LECS at our hospital since then.

The introduction of LECS required preoperative conferences and preparation for simultaneous laparoscopic and endoscopic procedures in the operating room. The importance of time-out and briefing has been discussed recently [7]. In this study, we hoped to make the importance of such attempts known to the public to promote patient safety and achieve smooth operation as an organization.

There have been several reports [8–14] of local resection as the standard technique for gastric SMTs. There have been reviews as well, especially of LECS. However, few papers have discussed how to choose the surgical method and the results at a single institution [15].

The upper stomach is a frequent site for gastric SMTs [16]. As stated, non-LECS was chosen for extraluminal and intramural lesions. In contrast, intraluminal lesions were treated using non-LECS procedures in 7 cases and LECS in 12 patients (Table 1). Only one patient was treated with non-LECS procedures for a lesion in the lesser curvature because it was a large tumor. The tumor was resected, and the mucosa was closed directly overlying the tumor site after removing the fatty attachment of the lesser curvature under laparoscopy. For tumors in the greater curvature, five patients each underwent non-LECS procedures and LECS. However, a detailed review showed that LECS was performed from 2014 to 2016, and recently, resection using a stapler transabdominally has been standardized; therefore, all recent patients underwent non-LECS procedures. Figure 2 shows how we localized the tumors. In the LECS group, we were able to select this less invasive surgery for patients who would otherwise have had to undergo laparotomy owing to the tumor location. Figure 3 shows the essential parts of the decision-making process for the selection of the optimal surgical procedure for gastric SMTs. We hope that this information will help clinicians choose the appropriate surgical procedure.

Table 2 shows the characteristics of the non-LECS and LECS patients in this study. One patient in the LECS group developed an abdominal abscess as a complication, which resulted in a postoperative length of stay of 57 days. The lesion was located on the posterior wall of the stomach, and gastric juice leakage was considered to have caused the abscess. Thereafter, we recognized the importance of intraperitoneal lavage before wound closure.

In Japan, full-thickness resection is the standard treatment for gastric SMTs. Endoscopic full-thickness resection has recently been reported for small gastrointestinal lesions that are less than 30 mm in size and for intraluminal lesions [17]. In our study, as intraluminal lesions were more common in the lesser and greater curvatures in the upper third of the stomach, we recommended LECS for lesions in the lesser curvature and non-LECS for lesions in the greater curvature. The development of new techniques may change treatment strategies in the future.

This study has limitations, as it was based on a single institution’s experience in Japan. Therefore, further study is needed.

## Conclusions

We reported the surgical procedures performed for gastric SMTs in our hospital over a 7-year period. We reported for the first time that the site and type of growth of a gastric SMT as determined preoperatively can be used to determine whether LECS or non-LECS is the optimal surgical approach. It is essential to make full use of the multiple techniques reported in this article and to evaluate the tumor location preoperatively to avoid excess or insufficient resection.

## Data Availability

Due to the nature of this research, this study did not agree that their data should be shared publicly, so supporting data is unavailable.
